# Dietary Choices and Habits during COVID-19 Lockdown: Experience from Poland

**DOI:** 10.3390/nu12061657

**Published:** 2020-06-03

**Authors:** Aleksandra Sidor, Piotr Rzymski

**Affiliations:** 1 Faculty of Medicine, Poznan University of Medical Sciences, 60-812 Poznań, Poland; aleksandrasidor13@o2.pl; 2 Department of Environmental Medicine, Poznan University of Medical Sciences, 60-806 Poznań, Poland

**Keywords:** COVID-19, SARS-CoV-2, dietary habits, alcohol use, smoking, quarantine, social distancing

## Abstract

The outbreak of coronavirus disease (COVID-19) in late December 2019 in China, which later developed into a pandemic, has forced different countries to implement strict sanitary regimes and social distancing measures. Globally, at least four billion people were under lockdown, working remotely, homeschooling children, and facing challenges coping with quarantine and the stressful events. The present cross-sectional online survey of adult Poles (*n* = 1097), conducted during a nationwide quarantine, aimed to assess whether nutritional and consumer habits have been affected under these conditions. Over 43.0% and nearly 52% reported eating and snacking more, respectively, and these tendencies were more frequent in overweight and obese individuals. Almost 30% and over 18% experienced weight gain (mean ± SD 3.0 ± 1.6 kg) and loss (−2.9 ± 1.5 kg), respectively. Overweight, obese, and older subjects (aged 36–45 and >45) tended to gain weight more frequently, whereas those with underweight tended to lose it further. Increased BMI was associated with less frequent consumption of vegetables, fruit, and legumes during quarantine, and higher adherence to meat, dairy, and fast-foods. An increase in alcohol consumption was seen in 14.6%, with a higher tendency to drink more found among alcohol addicts. Over 45% of smokers experienced a rise in smoking frequency during the quarantine. The study highlights that lockdown imposed to contain an infectious agent may affect eating behaviors and dietary habits, and advocates for organized nutritional support during future epidemic-related quarantines, particularly for the most vulnerable groups, including overweight and obese subjects.

## 1. Introduction

The novel coronavirus disease (COVID-19), induced by SARS-CoV-2 and firstly reported in late December 2019 in Wuhan, China, quickly became an emerging, rapidly evolving situation, spreading inevitably outside China and the Asian continent, and it was declared a pandemic in March 2020 [1,2,3]. Under these circumstances, different countries confirming their first cases began to implement a strict hygiene regime and eventually imposed city-wide and national lockdown measures. As a result, an estimated 4 billion people were forced to quarantine themselves at home. As learned from experiences with SARS, quarantine, particularly mandatory, can result in a high prevalence of psychological distress, manifested most frequently by low mood and irritability [4], with emotional disturbance and exhaustion, anger, insomnia, post-traumatic stress, and depressive symptoms also reported [5,6,7,8,9]. The COVID-19 pandemic has received broad coverage from worldwide mass media, often applying click-bait techniques to generate higher revenue, and using references such as ‘killer virus,’ ‘deadly virus,’ ‘alarming spread,’ or ‘highly contagious disease’ [10,11,12]. Moreover, this particular pandemic has arisen in a time of global internet use and the increasing role of online social media in the spread of information, including unsupported claims and fake news often amplified by algorithms and deliberate individual actions [13,14]. All of this has the potential to further exacerbate the effect of mandatory quarantine on anxiety, fear, and panic. 

Therefore pandemic-related quarantine can be classified as a stressful event, and in general, such events are known to affect eating patterns [15]. Depending on whether the stress is acute or chronic, hypophagia or hyperphagia and binge-eating can be induced, both eventually resulting in a significant weight change [16,17,18]. Prolonged staying at home may also support eating palatable meals, snacking, and alcohol consumption [19]. It may further affect individual choices to cook more or buy prepared food more often. A healthy balanced diet is an integral part of a personal risk management strategy during pandemics, such as the one of COVID-19 [20]. It does not entirely prevent the infection, although it may play a profound role in the host response to an infectious agent. Various macro-, micro-, and phytonutrients have immunomodulatory effects and are required for immunocompetence, whereas nutritional deficiencies are linked to higher host susceptibility to viral infection and a more severe clinical course of disease [21,22]. At the same time, nationwide lockdown due to disease outbreak may potentially alter dietary habits, as it forces the majority of individuals to stay at home for a prolonged period of time, often with unlimited access to food and lower physical activity. This is of particular concern in the case of individuals with pre-existing nutritional issues. 

To address the above-discussed issues, the present cross-sectional online survey study explored the dietary habits during the nationwide COVID-19-related quarantine in Poland. The frequency of food consumption, snacking, alcohol use, and smoking, as well as weight change, were assessed. Additionally, public fears of contracting the infection while shopping for groceries and during direct contact with food were also evaluated. The first case in the country was confirmed on 4 March 2020, school and university closure was imposed on 11 March; on 15 March, borders were closed to foreigners; from 24 March, a nationwide lockdown was imposed; on 20 April, the ban on the recreational use of forests and parks was lifted; on 4 May, hotels and shopping centers were permitted to reopen; and since 6 May, daycare centers and kindergartens have been allowed to welcome their pupils again. In other words, for the majority of Poles, the stay-at-home order encompassed six weeks. For selected groups of individuals, this period was, however, longer considering that schools and universities were the first to shut and, at the moment of writing, remain closed. Understanding how this situation could affect dietary habits is essential for an understanding of the role that nutritionists and dietitians can play in epidemic-related quarantine in the future. 

## 2. Material and Methods

To explore the effects of nationwide quarantine on nutritional and consumer habits in Poland, an anonymous, online survey based on a self-designed, structured questionnaire was conducted (Supplementary Materials). Such online research is a recommended approach to swiftly reach a specific group of subjects, ensuring their safety under pandemic conditions [23,24]. The questionnaire was made available via online social media for a period of two weeks between 17 April and 1 May. During this time, the number of confirmed COVID-19 cases in Poland increased from 8379 to 13,105 according to data available at the COVID-19 Dashboard by the Center for Systems Science and Engineering at Johns Hopkins University [25]. 

The inclusion criteria for the study were Polish nationality, age ≥ 18 years old, and female or male gender. Individuals working on a regular basis during the lockdown were excluded. These criteria were verified by answers given to the corresponding survey questions. 

The employed questionnaire aimed to assess:

1. Whether quarantine resulted in increased food consumption, snacking, and cooking;
2. Daily number of consumed meals and snacks during quarantine;
3. Frequency of consumption of selected food products (fresh vegetables and fruits, legumes, grain products, meat products, dairy, fast-foods, sweets, and salty snacks) during the quarantine;
4. Frequency of breakfast consumption during quarantine;
5. Observed weight change during quarantine;
6. Alcohol consumption in the general population and in individuals addicted to alcohol during quarantine;
7. Smoking frequency in smokers during quarantine;
8. Level of fears of contracting SARS-CoV-2 during grocery shopping and through contact with food products.

The frequency of consumption of each product was assessed using categorical variables defined as more than once a day, daily, a few times per week, once a week, once a month, occasionally, and never [26]. Since the study was conducted during the challenging time of the pandemic-related lockdown, the food frequency assessment was simplified to avoid the negative effect of the length of the questionnaire on the response rate [27,28]. The demographic data on each surveyed individual included age, gender, place of living (urban or rural), level of education (primary, secondary, tertiary, or vocational), and BMI (calculated from reported weight and height). Dichotomous questions were applied to identified smokers and those suffering from alcohol addiction. 

The data were elaborated with Statistica v. 13.1 (StatSoft Inc., Tulsa, OK, USA). Since the variables expressed in the interval scale (age and BMI) did not meet the assumption of Gaussian distribution (Shapiro–Wilk test; *p* < 0.05), nonparametric methods were used for statistical elaboration. 

To analyze the differences in age and BMI between two or more independent groups, the Mann–Whitney U test or Kruskal–Wallis analysis of variance (ANOVA) with Dunn’s post-hoc method were used, respectively. To assess whether consumption of each of considered food product varied between groups, the categorical frequencies were transformed into a 7-point scale. The correlations between age and BMI and weight change were assessed with Spearman’s correlation coefficient. A value of *p* < 0.05 was considered statistically significant.

## 3. Results and Discussion

### 3.1. Demographic Characteristics

The survey was completed by 1097 Poles. The demographic breakdown of the studied population is presented in Table 1. Most of the surveyed individuals were aged 18–25 years, female, inhabited urban areas, had a normal BMI, and had completed tertiary education. Smoking and alcohol addiction was declared by 155 (14.1%) and 14 (1.3%) subjects, respectively (Table 1). 

### 3.2. Dietary Patterns

In general, 43.5% of surveyed individuals reported eating more during quarantine, and 51.8% admitted to snacking between meals more frequently. The most frequent number of meals per day during quarantine in the studied group was three (30.3%) and four (39.3%), while in case of snacks, it was one (28.3%) and two (36.1%). Compared to the group not reporting modifications, increased food consumption and snacking was reported by individuals with higher BMI (mean ± SD, 22.9 ± 4.6 vs. 24.1 ± 5.1 and 22.0 ± 4.6 vs. 23.9 ± 5.0, respectively; Mann–Whitney U, *p* < 0.01 in all cases). Cooking more often during quarantine was declared by 62.3% of surveyed individuals and was not differentiated by BMI (Mann–Whitney U test, *p* > 0.05). The observed frequencies of increased food consumption, snacking, and cooking in different BMI groups are summarized in Table 2. Eating and snacking more during quarantine was most frequent in obese subjects. 

Changes in food consumption, snacking, and cooking during the quarantine were not differentiated by gender, place of living, education level, occupation status (Pearson’s χ^2^, *p* > 0.05 in all cases), or age (Mann–Whitney U test, *p* > 0.05). The present study clearly demonstrates that quarantine may pose a significant dietary risk, particularly for overweight and obese individuals. In general, these groups are known to display more problematic eating behaviors, including food consumption in the absence of hunger and frequent overeating [29], which may be further promoted during quarantine due to prolonged stay at home with often unlimited access to food. It is also known that an increase in snacking, as seen in the present study across all BMI groups, can lead to an increase in fat mass and percentage [30]. However, previous research has shown that individuals with a higher weight tend to snack significantly more often in the evening, and this is more detrimental to healthy weight compared to snacking at other times of day [31]. 

The majority of people surveyed, 65.5%, reported eating breakfast every day; eating it almost every day was declared by 20.4%, sometimes by 8.5%, practically never by 20.4%, and never by 1.2%. These frequencies were not differentiated across BMI and age groups nor by gender, place of living, and occupation status (Pearson’s χ^2^, *p* > 0.05 in all cases). However, eating breakfast every day was more often seen in individuals with higher education (72.3%) than with vocational (66.7%), secondary (58.9%), and primary (42.9%) education level (Pearson’s χ^2^, *p* < 0.001). It is now well evidenced that skipping breakfast can be associated with an increased risk of type 2 diabetes [32], heart disease [33], and mortality from cardiovascular disease [34]. It is likely that this information, also reported by various health-promoting and diet-associated websites, online blogs, and books, more often reaches individuals with higher education. The challenge that remains is to reach out to the groups that may not actively seek evidence-based information, and particular attention in this regard should be paid during a pandemic and associated quarantines and nationwide lockdowns. Moreover, it should be stressed that the nutritional quality of the eaten breakfast is likely to be of higher importance than only eating breakfast on a daily basis [35].

The present study also examined the frequency at which particular food products were consumed during quarantine (Table 3). As observed, nearly one-third of those surveyed did not consume fresh vegetables and fruits on a daily basis, while the same proportion admitted to consuming sweets at least every day. 

Significant associations between BMI and frequency of consumption of selected food products during quarantine were observed (Spearman’s Rs, *p* < 0.05 in all cases), i.e., with vegetables and fruits (Rs = −0.19), legumes (Rs = 0.11), fast-foods (Rs = 0.19), meat (Rs = 0.24), dairy (Rs = 0.10), and coffee (Rs = 0.17). Among all BMI groups, the obese subjects had the lowest frequency of consuming vegetables, fruits, and legumes on a daily basis (58.5 and 13.8%), and the highest frequency of consuming fast-foods (3.2%), meat (40.4%), and dairy (54.2%). 

There was also a correlation between the age of surveyed individuals and the frequency of consuming meat (Rs = 0.32), sweets (Rs = 0.10), vegetables and fruits (Rs = −0.10), legumes (Rs = −0.11), and coffee (Rs = 0.34) (Spearman’s Rs, *p* < 0.05 in all cases). The group aged >45 displayed the lowest frequency of consuming vegetables and fruits (63.0%), legumes (15.3%), dairy (37.0%), and sweets (25.9%) on a daily basis, and the highest frequency of consuming meat (38.9%) and coffee (88.9%). 

Surveyed men consumed meat and instant products more often in comparison to women (Mann–Whitney U, *p* < 0.05), the frequency of men consuming them on a daily basis was higher—37.7 vs. 20.3% and 5.7 vs. 0.2%, respectively. No difference between the consumption frequency of any food product was seen between inhabitants of urban and rural areas (Mann–Whitney U, *p* < 0.05 in all cases). Neither occupational status nor education level differentiated surveyed individuals in this respect (Kruskal–Wallis, *p* < 0.05 in all cases). 

In line with expectations, the present study shows that during the outbreak-related lockdown, the eating behaviors in individuals ordered to follow stay-at-home advice are often modified and that a general tendency to consume more food can be seen. Although such lockdowns are only imposed temporarily in order to decrease the transmission of an infectious agent in population, they may have long-lasting effects on humans. For instance, previous studies have demonstrated that prolonged quarantine might affect mental health by increasing symptoms of post-traumatic stress disorder and depression [6]. Whether a nationwide quarantine may also result in the reinforcement of modified dietary habits and contribute to dietary-related health effects in the longer perspective remains subject to study. There is, however, in vivo evidence that social isolation is related to increased food consumption and the associated development of obesity and type 2 diabetes [36]. Prolonged stay at home during quarantine enables unlimited access to food and may, therefore, cause a perturbation of time-restricted feeding, which is known to support robust metabolic cycles and has a protective role in dysmetabolism and obesity [37]. 

Importantly, the present study further shows that those with increased BMI, and obese subjects in particular, are most prone to quarantine-related adverse dietary modification resulting in increased food consumption and snacking; the lowest frequency of fruit, vegetable, and legume consumption on a daily basis; and the greatest tendency to consume meat, sweets, salty snacks, and fast foods every day. These results clearly show the need for appropriate dietary support for overweight and obese subjects during lockdowns as this group has increased vulnerability to infections and their severity [38,39,40]. Under the pandemic scenario, the management of overweight or obesity is even more challenging. Therefore, it is pivotal for individuals affected by higher BMI to adhere to individual risk management strategies that should also include a well-balanced diet [20]. In particular, the consumption of vegetables and fruits may be beneficial in this regard due to accumulating evidence of the antioxidant and anti-inflammatory effects of their components: vitamin C, carotenoids, B vitamins, and polyphenols such as flavonoids, phenolic acids, stilbenes, and lignans [41,42,43,44]. There is also some indication that food legumes may reveal the anti-inflammatory action that is attributed to lectins and peptides [45]. In general, observational studies show that adherence to plant-based diets is associated with lower levels of inflammation markers, e.g., C-reactive protein, fibrinogen, and interleukin-6, compared to omnivorous diets [46,47,48]. This is further supported by interventional trials showing that plant-based dietary approaches are associated with lower scores of Dietary Inflammatory Index and improved inflammatory markers in subjects with high BMI [49,50,51,52]. In contrast, the present study demonstrates that overweight and obese subjects during quarantine tend to eat less vegetables and fruits while revealing the most frequent consumption of meat, dairy, and fast-foods. A fast food diet and high consumption of meat products, particularly red and processed red meat, has been associated with pro-inflammatory effects [53,54]; therefore, it seems reasonable that their consumption should not be preferred to vegetables, fruits, and legumes, particularly during a pandemic of an infectious agent, and especially in groups with increased vulnerability. One should, however, note that the declared consumption of fresh vegetables and fruits was generally low in the studied group—only 67.2% admitted to consuming them on a daily basis. This worrisome observation indicates that nutritional awareness among Poles is not sufficient and requires further attention in order to promote healthy eating choices and other nutrition-associated behaviors. 

### 3.3. Reported Change of Weight

**Figure 1:**
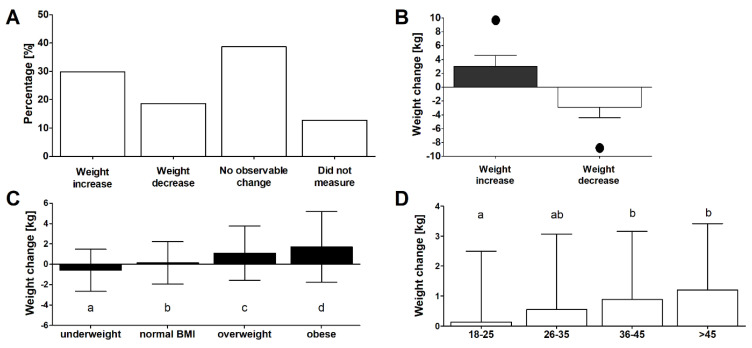
(**A**) Reported trends in weight change during COVID-19 lockdown in the surveyed group (*n* = 1097). (**B**) Mean ± SD of experienced weight increase or decrease (black dot represents the maximum value of change reported in the surveyed group). (**C**) Weight change in different BMI groups. (**D**) Weight change in different age groups. Different lowercase letters denote significant differences between groups (Dunn’s test after Kruskal–Wallis analysis of variance (ANOVA), *p* < 0.05).

Changes in dietary patterns during quarantine can potentially result in weight change as a result of lower physical activity, changes in food consumption, and the stress associated with adapting to a new situation. In the present study, 29.9 and 18.6% of those surveyed reported an increase and decrease of weight, respectively (Figure 1A). Specific details on observed weight changes are presented in Figure 1B—the maximum declared weight gain and loss during the quarantine period were 10 and 9 kg, respectively. The weight change was correlated with BMI (Rs = 0.21, *p* < 0.05) and age (Rs = 0.15, *p* < 0.05), with significant gains observed particularly in overweight and obese subjects (Figure 1C), and those aged 35–45 and >45 years old (Figure 1D). No association between weight gain and education level, place of living, or occupation status was seen (Kruskal–Wallis ANOVA, *p* > 0.05 in all cases). Moreover, no significant difference in changes in weight was observed between women and men (Mann–Whitney U, *p* > 0.05).

Increased weight, and particularly obesity, has been associated with a more severe clinical course of COVID-19 and risk of fatality [38,39,40]. Age has also been observed to modify disease severity. As reported for mainland China, the proportion of patients infected with SARS-CoV-2 requiring hospitalization increased from 1.0% and 4.3% for subjects aged 20–29 and 30–39, respectively, to 4.3%, 8.2%, 11.8%, 16.6%, and 18.4% for individuals aged 40–49, 50–59, 60–69, 70–79, and ≥80, respectively [55]. Therefore, considering vulnerability, older and obese subjects are among those who should particularly adhere to social distance measures. On the other hand, the present study shows that these groups are most prone to weight gain during quarantine, most likely owing to lower physical activity and, as discussed in the previous subsection, changes in dietary pattern, e.g., increased frequency of snacking. At the same time, underweight subjects tended to lose weight. Altogether, these observations lead to the general conclusion that quarantine can reinforce pre-existing alterations in body weight and potentially magnify BMI-related health issues. This, in turn, calls for organized nutrition support during future lockdowns that could be achieved through the engagement of health authorities, social media campaigns, and remote dietitian services. 

### 3.4. Alcohol Consumption and Smoking

Both alcohol consumption and smoking can potentially increase vulnerability to SARS-CoV-2 infection and worsen the clinical course of COVID-19. Chronic alcohol exposure has a complex and adverse effect on host response [56], including the mechanisms of innate and adaptive immunity, and is known to increase susceptibility to viral infection. 

Prolonged stays at home can potentially affect alcohol consumption. As evidenced in mammals, long isolation results in increased stress levels [57], while stress is a well-established risk factor for alcohol misuse. Although, in the present study, the majority of those surveyed (77%) did not report any increase in drinking during quarantine and 8.3% were uncertain whether their consumption was affected, 14.6% reported a rise in alcohol consumption. One should, therefore, consider that during lockdowns, some individuals are more prone to alcohol overuse. Consequently, this may lead to the onset of alcohol use disorder among subjects at higher risk. No association between a change in the frequency of alcohol consumption and age and BMI of surveyed individuals was identified (Mann–Whitney U, *p* > 0.05 in both cases). Gender, education level, occupational status, and place of living also did not differentiate this parameter (Pearson’s χ^2^, *p* > 0.05 in all cases).

Importantly, an increase in alcohol use during quarantine was found to be more frequent in individuals who declared themselves to be addicted compared to those perceiving themselves as non-addicts (64.0 vs. 14.0%; Pearson’s χ^2^, *p* < 0.001,). Even though the number of self-identified alcohol addicts in the study was low (*n* = 14), this highlights the need to provide assistance and support for this group, and in all likelihood other chemical abusers, during epidemic-related lockdowns. As postulated, public health warnings regarding excessive alcohol use during quarantine should also be issued to protect more vulnerable subjects [58].

In the group of smokers, 40% reported that their smoking frequency did not change during quarantine, 14.8% were uncertain if their smoking habit was affected while as many as 45.2% reported smoking more. Age and BMI of smokers were not associated with a change in smoking frequency (Mann–Whitney U, *p* > 0.05 in both cases), and neither were gender, education level, nor place of living (Pearson’s χ^2^, *p* > 0.05 in all cases). However, an increase in smoking was more frequently seen in full-time workers (15.6%) than students (11.2%) or those who are unemployed (10.8%) (Pearson’s χ^2^, *p* < 0.01). The COVID-19 pandemic can be considered to be a stressful event, and it can be expected that individuals under nationwide lockdown are facing, at least temporarily, higher stress levels due to the need to adapt to the prolonged stay at home. These adaptations may be more challenging for full-time workers than students or unemployed subjects, as they face a need to switch to remote working, often under high pressure [59]. Smokers, in turn, often perceive cigarettes as a stress relief [60]. There is a piece of experimental evidence that smoking during unpleasant circumstances may decrease the level of arousal resulting in temporary stress relief, [61] although studies have also shown that smoking may eventually lead to generation or aggravation of negative emotional states, support adverse coping strategies, and increase the overall stress level [62,63,64]. Whether increased smoking during the lockdown may result in a higher consumption of cigarettes in the future remains unknown. 

### 3.5. Fears During Grocery Shopping and Food Contact

**Figure 2:**
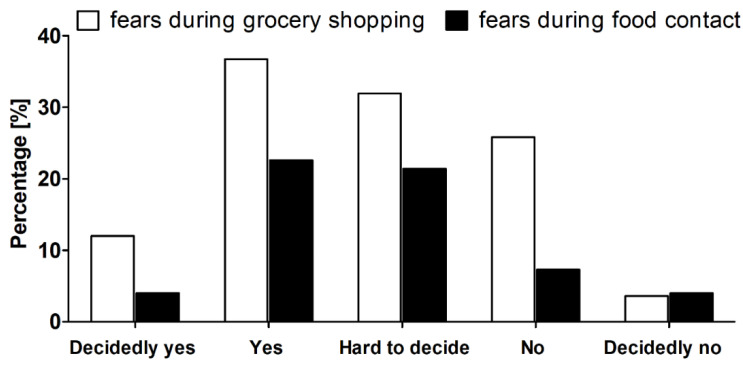
Reported fears over contracting SARS-CoV-2 during shopping for food and contact with food in the surveyed group (*n* = 1097).

The outbreak of COVID-19, its rapid global spread, and the restrictions imposed by the subsequent countries inevitably led to public fears and panic, magnified by a broad mass and social media coverage, misinformation, and pseudoscience. When the nationwide lockdown in Poland was imposed on 24 March 2020, the media broadly commented on the worsening situation in Italy, with 69,176 confirmed cases and 6820 deaths [25], adding to an already high level of anxiety. For the majority of Polish inhabitants, grocery stores became one of the few public places visited during the lockdown, while the use of face masks was made compulsory on 16 April. In other words, shopping for food could quickly become perceived as a primary activity that increased the risk of contracting SARS-CoV-2 infection. In line with this, 48.7% of the group surveyed in the present study revealed a fear of contracting SARS-CoV-2 during grocery shopping, whereas 31.9% could not decide on this matter (Figure 2). After excluding the latter group, women were observed to express this fear significantly more often than men (63.2 vs. 50.0%; Pearson’s χ^2^, *p* < 0.05). It was also more frequently present among individuals with a vocational (81.2%) and tertiary (65.9%) education level than those with secondary (59.2%) and primary (35.7%) (Pearson’s χ^2^, *p* < 0.05). In turn, occupation status and place of living did not significantly diversify the surveyed subjects in this regard. 

One should note that fear during grocery shopping may not be undesirable per se, as it can potentially lead to better adherence to mandatory as well as voluntary preventive measures. It is widely recognized that a lower education level is associated with worse health knowledge and awareness, and a higher risk of contracting the viral infection [65,66,67]. This may explain the lowest frequency of fear observed by the present study in adults with primary education, and may potentially reflect more careless, non-preventive behaviors during grocery shopping, although such an aspect was beyond the scope of this research. In turn, the difference found in this respect between women and men is in line with the evidence that the former are generally more prone to fear and anxiety [68]. Previous surveys have indicated that women report a higher rate of concern over COVID-19 and perceive more risks [69,70], despite findings that men are more prone to experiencing a severe course and fatal outcome of the disease [71]. Again, this concern and fear may result in better adherence of women to prevention recommendations, e.g., hand hygiene, mask-wearing, and physical distancing—a phenomenon observed during SARS and COVID-19 outbreaks [72,73]. 

Additionally, 27.5% of the surveyed reported a fear of contracting the SARS-CoV-2 infection when having direct contact with food (Figure 2). No associations in this regard with gender, education level, place of living, and occupation status were observed (Pearson’s χ^2^, *p* > 0.05). There is no evidence that this is a source or transmission route of SARS-CoV-2, and this has been highlighted by different authorities, including the European Food Safety Authority [74]. However, it is plausible that this fear arose from concerns that an infectious virus could be present on the surface of both non-prepacked and packed food. As reported experimentally under stable conditions of room temperature, viable SARS-CoV-2 was detectable up to 72 h on a plastic surface [75], while plastics remain the most common and most wide-ranging materials used for food packaging [76]. 

### 3.6. Limitations

Although this study provides an insight into how the epidemic-related lockdown can affect dietary patterns and weight, there are some limitations that also need to be underlined. Firstly, research based on an anonymous and online survey excludes the possibility of verifying the data on objective grounds. BMI was not measured directly before and after the quarantine but declared by the surveyed individuals. Therefore, it must be treated more as a rough estimate, not an exact value. These limitations were, however, impossible to overcome if one considers the challenges of conducting such a study during a nationwide quarantine. It should also be stressed that the subset of men was under-represented, which is often the case in voluntary survey investigations. Some other analyzed groups were also not homogenous, and the selected ones, such as individuals declaring an addition to alcohol, were low in number. Therefore, the possible non-response bias should be taken into account when interpreting the survey results, particularly in the context of male gender and individuals addicted to alcohol; they should be treated as a series of cases rather than true groups. Moreover, the present investigations used a simplified approach to provide a general summary of the frequency of consumption of foodstuffs, and the consumption of particular types of each food product category (e.g., meat, vegetables, etc.) was not distinguished. This was, however, done to avoid the negative effect of questionnaire length and content on the response rate [27,28], which could particularly be present if one considers the challenging circumstances under which the survey was conducted. Finally, while the present study provides an overview of dietary habits and modifications during quarantine, its results cannot be interpreted in the context of long-term effects, as this was not an aim. All this suggests the need for cautious data interpretation and calls for further studies in this field. 

## 4. Conclusions

The present study indicates that during a pandemic-associated nationwide quarantine, a significant percentage of individuals can experience modification of dietary habits, manifested by eating and snacking more, and weight change. What is of particular concern is the finding that overweight and obese subjects are most prone to these modifications while at the same time they tend to eat vegetables, fruits, and legumes less frequently, and salty foods, meat, and dairy more often. Considering that higher BMI is associated with increased severity of COVID-19 as well as other infections, a strategy to decrease the potentially detrimental dietary effects of lockdown in this group should be considered. Importantly, a tendency for weight loss in underweight subjects is also worrisome and requires attention. Moreover, the study demonstrates that nationwide quarantine can result in increased alcohol consumption, at least in Poland, and that this may also be experienced by individuals addicted to alcohol. Again, the proactive development of strategies mitigating this phenomenon must be stressed. Future research is required to understand whether the COVID-19-related lockdown has resulted in long-term reinforcement of adverse dietary habits and associated health issues. 

## Figures and Tables

**Table 1:** Demographic breakdown of surveyed participants (*n* = 1097).

**Age (years) mean ± SD (min–max)** 18–25, *n* (%) 26–35, *n* (%) 36–45, *n* (%) >45, *n* (%)	27.7 ± 9.0 (18–71) 588 (53.6) 310 (28.3) 144 (13.1) 54 (4.9)
**Gender** Female, *n* (%)/Male, *n* (%)	1043 (95.1)/54 (4.9)
**Weight** (kg) mean ± SD (min–max) **Body Mass Index** (kg/m^2^) mean ± SD (min–max) Underweight (<18.5), n (%) Normal weight (18.5–24.9), n (%) Overweight (25.0–29.9), n (%) Obesity (≥30.0), n (%)	66.0 ± 14.5 (40–140) 23.5 ± 4.8 (14.4–57.8) 87 (7.9) 699 (63.7) 217 (19.8) 94 (8.6)
**Occupation** Unemployed, n (%) Student, n (%) Full-time worker, n (%)	110 (10.0) 518 (47.2) 469 (42.8)
**Place of living** Urban, *n* (%)/Rural, *n* (%)	881 (80.3)/216 (19.7)
**Education** Primary, *n* (%) Secondary, *n* (%) Tertiary, *n* (%) Vocational, *n* (%)	35 (3.2) 474 (43.2) 567 (51.7) 21 (1.9)
**Declared addictions** Alcohol, *n* (%)/Smoking, n (%)	14 (1.3)/155 (14.1)

**Table 2:** The frequency (%) of increased food consumption, snacking, and cooking in the surveyed group (*n* = 1097) across the BMI groups.

	Underweight	Normal BMI	Overweight	Obese	Pearson’s χ^2^
Eating more	40.7	30.6	48.8	55.3	*p* < 0.05
Snacking more	46.5	50.1	55.3	61.7	*p* < 0.05
Cooking more	63.3	62.1	62.6	63.3	*p* > 0.05

**Table 3:** The frequency of consumption of particular foods during quarantine in Poland in the surveyed group (*n* = 1097).

	>1 per Day	Once per Day	Few Times per Week	Once per Week	Once per Month	Occasionally	Never
	Percentage of Surveyed [%]						
Vegetables and fruits	25.1	42.1	25.5	5.0	0.7	1.0	0.5
Legumes	3.6	16.8	40.2	20.7	11.4	5.5	1.9
Grain products	19.4	44.8	26.1	6.1	1.5	1.4	0.7
Meat products	3.1	18.0	28.5	7.0	1.6	4.8	36.8
Dairy	11.0	38.1	30.3	8.8	2.6	3.6	5.7
Fast-foods	0.3	0.7	6.7	12.8	27.8	28.3	23.4
Instant products	0.0	0.2	2.8	5.7	41.5	0.0	39.2
Sweets	6.7	26.1	36.6	17.7	5.2	4.9	2.8
Salty snacks	1.5	6.3	22.6	25.3	19.8	13.7	10.8
Coffee	27.7	30.1	10.4	5.7	2.9	6.1	17.1
Tea	37.1	29.6	17.4	5.3	2.6	4.6	3.4

## Supplementary Materials

The following are available online at https://www.mdpi.com/2072-6643/12/6/1657/s1, Translated Questionnaire.
