# Supplementary material for: Dietary Choices and Habits during COVID-19 Lockdown: Experience from Poland
Source: Nutrients. 2020 Jun 3;12(6):1657. doi: 10.3390/nu12061657 (PMC7352682; doi:10.3390/nu12061657)
Supplement: Supplementary file 1 [file nutrients-12-01657-s001.pdf]

TRANSLATED QUESTIONNAIRE

Did you consume more food than usual during quarantine?

a) decidedly yes b) yes c) hard to decide d) no e) decidedly no

Please indicate the number of meals eaten per day during quarantine:

a) one b) two c) three d) four e) five f) six or more

Did you snack more frequently than usual during quarantine?

a) decidedly yes b) yes c) hard to decide d) no e) decidedly no **Please indicate the number of snacks eaten per day during quarantine:**

a) none b) one c) two d) three e) four or more

Did you cook more often than usual during quarantine?

a) decidedly yes b) yes c) hard to decide d) no e) decidedly no

How often did you eat breakfast during quarantine?

a) every day b) almost every day c) sometimes d) practically never e) never

Please estimate the frequency of consumption of particular foods during COVID-19 quarantine:

	>1 per day	Once per day	Few times per week	Once per week	Once per month	Occasionally	Never
Vegetables and fruits							
Legumes							
Grain products							
Meat products							
Dairy							
Fast-foods							
Instant products							
Sweets							
Salty snacks							
Coffee							
Tea							

Did you notice any changes in your body weight during quarantine?

a) weight increase b) weight loss c) no change in weight d) did not measure weight

If you noticed any changes in your body weight during quarantine, please indicate the estimated difference (in kg): ….

Do you smoke cigarettes?

a) yes b) no

If so, did you observe a tendency to smoke more during quarantine?

a) decidedly yes b) yes c) hard to decide d) no e) decidedly no

Did you drink more alcohol during quarantine?

a) decidedly yes b) yes c) hard to decide d) no e) decidedly no

Are you addicted to alcohol?

a) yes b) no

Please indicate whether you fear contracting coronavirus during grocery shopping?

a) decidedly yes b) yes c) hard to decide d) no e) decidedly no

Please indicate whether you fear contracting coronavirus when having direct contact with food?

a) decidedly yes b) yes c) hard to decide d) no e) decidedly no

Please indicate which of the following describes you best during COVID-19 quarantine:

a) I work full-time remotely b) I work with no changes, not remotely c) I am a university student d) I am unemployed d) I work regularly

Your age (in years): …

Your weight (in kg): ….

Your height (in cm): ….

Your gender:

a) female b) male

Your place of living:

a) urban ≥250,000 residents b) urban 50,000–200,000 residents c) Urban 50,000–100,000 residents d) urban < 50,000 residents e) rural area

Your education level:

a) primary b) secondary c) tertiary d) vocational
